# Editorial: Interspecies Interactions Within Fermented Food Systems and Their Impact on Process Efficiency and Product Quality

**DOI:** 10.3389/fmicb.2022.902116

**Published:** 2022-04-22

**Authors:** Brian Gibson, Jian Zhao, Rosane Freitas Schwan

**Affiliations:** ^1^Chair of Brewing and Beverage Technology, Technical University of Berlin, Berlin, Germany; ^2^Food Science and Technology Group, University of New South Wales, Kensington, NSW, Australia; ^3^Department of Biology, Universidade Federal de Lavras, Lavras, Brazil

**Keywords:** fermentation, interspecies interactions, food and beverage, efficiency, safety, quality

Food is an integral part of human culture. Aside from its nutritional role, it connects us on a daily basis through the act of sitting and eating together, it contributes to feelings of shared identity in populations, it is an essential component of many formative events in our lives, and is a gateway to experiencing new cultures.

While the influence of food in defining human culture is well-understood, there is a growing appreciation of the role of microbial cultures in defining the properties of food. Archaeological evidence points to the use of fermentation over many thousands of years for the production of a range of foods and beverages. Fermentation serves to make foods more digestible, longer-lasting, safer, and more palatable. For our species, the role of fermentation in food production has arguably been as influential as the role of fire for cooking food.

For much of our history, these fermentation processes have been carried out by complex assemblages of organisms, with the use of single strains for fermentation of bread, beer, wine, etc. being a relatively recent development. We have however been seeing a growing interest in the use of microbial communities in food production. In the western world, this revival has been driven in part by the popularity of craft beer, natural wine, fermented non-alcoholic beverages (kefir, kombucha), and sourdough bread. In other parts of the world, complex cultures in fermentation represent continuation of tradition rather than revival.

This Research Topic from Frontiers in Microbiology aims to highlight the beneficial role of microbial complexity in driving fermentation processes and influencing food quality. The issue features ten articles describing investigations into complex microbial fermentation for the production of food, feed, and beverages. Contributions were received from Africa, Asia, Australia, Europe, and South America—highlighting the global importance of fermentation. Demonstrated benefits of particular communities during fermentation include improved flavor and color, greater safety, increased nutritional value, faster fermentation rates, reduced alcohol content, and better industrial applicability.

One of the most direct impacts of fermentation is the creation of specific flavor profiles. We see this in the work of Elhalis et al., who show improved sensory properties of coffee after fermentation with the yeast *Pichia Kudriavzevii*. Likewise, Köhler et al. note how specific yeast and bacteria combinations can improve the flavor of water kefir. Liu et al. describe the relationship between bacterial composition and flavor profile of Xifeng liquor (a spirit prepared from fermented sorghum).

Palatability of foods is not the only factor influenced by fermentation. Benefits extend also to improved digestibility and safety. Su et al. for example, note how fermentation of corn by-products with *Saccharomyces* yeast and lactic acid bacteria can improve their suitability for use as feed. Dahunsi et al. provide a comprehensive account of how the presence of lactic acid bacteria within fermentative microbial consortia have a critical role in ensuring the safety and shelf-life of many fermented African foods. Likewise, Huang et al. describe the metabolites produced by the yeast *Debaryomyces hansenii* can prevent the growth of contaminant molds during Danish cheese production.

The composition of species in a fermenting culture is strongly impacted by the environment, a fact that must be considered when trying to control or direct fermentation processes. The effect of environment is clearly seen in the study of Martinez et al. who describe how coffee fermentation is influenced by altitude, and how this is associated with changes in the natural microbiota. Substrate also influences the development of populations, as illustrated by Liu et al. who show how different microbial communities develop depending on the type of sorghum used in Xifeng liquour production. Su et al. likewise show how communities develop differently in different by-products of the corn starch industry. These, and other studies described in this Research Topic, demonstrate the power of high-throughput sequencing in studying microbial communities. This is particularly important for dynamic, complex or under-researched communities. This is exemplified in the studies on dark tea fermentation by Yan et al. or the work of Atter et al. on Ghanian cereal fermentation.

Despite the advantages of complex fermentations with respect to product quality, it should be noted that industrial application of such cultures is not without complications. More complexity may lead to less consistency in fermentation processes. The controlled use of cultures, e.g., supplementing natural microbial consortia with specific species, or rationalizing consortia so that they contain only the keystone species, represents a compromise in this respect. Examples are seen here in the work on cheese production by Huang et al. coffee fermentation by Elhalis et al. and water kefir production by Köhler et al.

Complex fermentations demonstrate great potential for improving food process efficiency, enhancing food quality, and increasing diversity of available foods and beverages. It is clear however that our ability to fully exploit such fermentations is limited by insufficient knowledge of how individuals in mixed populations interact to influence each other and their environment. It is our hope that this Research Topic represents a step forward in our understanding of these complex systems, and will to some extent facilitate the efficient production of a range of high quality food products in the future.

## Author Contributions

All authors listed have made a substantial, direct, and intellectual contribution to the work and approved it for publication.

## Conflict of Interest

The authors declare that the research was conducted in the absence of any commercial or financial relationships that could be construed as a potential conflict of interest.

## Publisher's Note

All claims expressed in this article are solely those of the authors and do not necessarily represent those of their affiliated organizations, or those of the publisher, the editors and the reviewers. Any product that may be evaluated in this article, or claim that may be made by its manufacturer, is not guaranteed or endorsed by the publisher.

